# A GWAS approach identifies *Dapp1* as a determinant of air pollution-induced airway hyperreactivity

**DOI:** 10.1371/journal.pgen.1008528

**Published:** 2019-12-23

**Authors:** Hadi Maazi, Jaana A. Hartiala, Yuzo Suzuki, Amanda L. Crow, Pedram Shafiei Jahani, Jonathan Lam, Nisheel Patel, Diamanda Rigas, Yi Han, Pin Huang, Eleazar Eskin, Aldons. J. Lusis, Frank D. Gilliland, Omid Akbari, Hooman Allayee

**Affiliations:** 1 Departments of Molecular Microbiology and Immunology, Keck School of Medicine, University of Southern California, Los Angeles, California, United States of America; 2 Department of Preventive Medicine, Keck School of Medicine, University of Southern California, Los Angeles, California, United States of America; 3 Department of Biochemistry & Molecular Medicine, Keck School of Medicine, University of Southern California, Los Angeles, California, United States of America; 4 Department of Computer Science and Inter-Departmental Program in Bioinformatics, University of California, Los Angeles, Los Angeles, California, United States of America; 5 Department of Human Genetics, David Geffen School of Medicine at UCLA, Los Angeles, California, United States of America; 6 Department of Medicine, David Geffen School of Medicine at UCLA, Los Angeles, California, United States of America; 7 Department of Microbiology, Immunology, and Molecular Genetics, David Geffen School of Medicine at UCLA, Los Angeles, California, United States of America; Stanford University School of Medicine, UNITED STATES

## Abstract

Asthma is a chronic inflammatory disease of the airways with contributions from genes, environmental exposures, and their interactions. While genome-wide association studies (GWAS) in humans have identified ~200 susceptibility loci, the genetic factors that modulate risk of asthma through gene-environment (GxE) interactions remain poorly understood. Using the Hybrid Mouse Diversity Panel (HMDP), we sought to identify the genetic determinants of airway hyperreactivity (AHR) in response to diesel exhaust particles (DEP), a model traffic-related air pollutant. As measured by invasive plethysmography, AHR under control and DEP-exposed conditions varied 3-4-fold in over 100 inbred strains from the HMDP. A GWAS with linear mixed models mapped two loci significantly associated with lung resistance under control exposure to chromosomes 2 (p = 3.0x10^-6^) and 19 (p = 5.6x10^-7^). The chromosome 19 locus harbors *Il33* and is syntenic to asthma association signals observed at the *IL33* locus in humans. A GxE GWAS for post-DEP exposure lung resistance identified a significantly associated locus on chromosome 3 (p = 2.5x10^-6^). Among the genes at this locus is *Dapp1*, an adaptor molecule expressed in immune-related and mucosal tissues, including the lung. *Dapp1*-deficient mice exhibited significantly lower AHR than control mice but only after DEP exposure, thus functionally validating *Dapp1* as one of the genes underlying the GxE association at this locus. In summary, our results indicate that some of the genetic determinants for asthma-related phenotypes may be shared between mice and humans, as well as the existence of GxE interactions in mice that modulate lung function in response to air pollution exposures relevant to humans.

## Author summary

The genetic factors that modulate risk of asthma through gene-environment (GxE) interactions are poorly understood, due in large part to the inherent difficulties in carrying out such studies in humans. To address these challenges, we used the Hybrid Mouse Diversity Panel to elucidate the genetic architecture of asthma-related phenotypes in mice and identify loci that are associated with airway hyperreactivity (AHR) under control exposure conditions and in response to diesel exhaust particles (DEP), as a model traffic-related air pollutant. In the absence of exposure, we identified two loci on chromosomes 2 and 19 for AHR. The locus on chromosome 19 harbors *Il33* and is syntenic to association signals observed for asthma at the *IL33* locus in humans. In response to DEP exposure, we mapped AHR to a region on chromosome 3 and used a genetically modified mouse model to functionally demonstrate that *Dapp1* is one of the genes underlying the GxE association at this locus. Collectively, our results support the concept that some of the genetic determinants for asthma-related phenotypes may be shared between mice and humans as well as the existence of GxE interactions in mice that modulate lung function in response to air pollution exposures relevant to humans.

## Introduction

Asthma is a complex disease characterized by chronic airway inflammation that affects over 300 million people worldwide [1]. It is generally accepted that susceptibility to asthma results from interactions between genetic risk factors and environmental exposures, such as allergens and air pollutants [2–4]. In this regard, particulate matter with diameter less than 2.5 μM (PM_2.5_) is a well-studied ambient air pollutant that has been linked to reduced lung development in children and risk of asthma [5]. Traffic emissions, including combustion products, are major sources of PM_2.5_ [6] that can penetrate deep into small lung airways where they trigger immune responses either alone or by acting as adjuvants for simultaneously inhaled aeroallergens [7–10]. However, not all individuals respond the same to such exposures and evidence suggests that genetic background can modulate differences in the response to PM_2.5_ and other ambient air pollutants [5, 6, 8, 11–13].

Estimates for the heritability of asthma have ranged between 35%-95% [14], suggesting a strong genetic component to risk of asthma. Genome-wide association studies (GWAS) in humans support this notion and have identified ~200 loci for asthma and related pulmonary phenotypes [15–35]. Interestingly, the genes located at these loci along with bioinformatic analyses provide compelling genetic evidence for a strong inflammatory component to asthma susceptibility [31, 35]. Despite the large numbers of loci identified to date, the risk alleles, most of which are common in the population, still only explain a small fraction (~7%) of asthma’s overall heritability [36, 37]. This observation implies either the existence of additional variants with smaller effect sizes, rare susceptibility alleles, and/or higher order interactions between genes and environmental factors. Furthermore, while identification of genes is one important step towards improving our understanding of asthma pathogenesis, it does not provide direct information about interactions between genetic risk factors and environmental triggers. This is compounded by the inherent difficulties of carrying out gene-environment (GxE) interaction studies in humans. For example, accurate exposure assessments, adequately powered sample sizes in which genetic, phenotypic, and exposure data are all available, and the heterogeneous nature of asthma itself pose significant practical and technical hurdles that have yet to be overcome. Thus, the complex GxE interactions that modulate risk of asthma remain unknown. However, a better understanding of these interactions may provide important novel insights into the biological mechanisms of asthma and potentially identify subgroups of individuals at higher risk [38].

To circumvent the challenges of genetic studies of asthma-related traits in humans, gene discovery efforts have leveraged naturally occurring variation in mice. A variety of strategies, including linkage mapping with crosses or association studies with small numbers of inbred strains, have identified several loci for airway hyperreactivity (AHR) under both unexposed conditions or after exposure to various allergens, such as ovalbumin or house dust mite (HDM) [39]. In this regard, we have developed a panel of inbred mouse strains, termed the Hybrid Mouse Diversity Panel (HMDP) [40], that has been used to identify loci for a number of complex traits relevant to human diseases [41–45]. Furthermore, the renewable nature of the HDMP, where multiple subjects of the same genotype can be studied, has been proven useful for GxE studies with a diverse array of exposures, including inflammatory responses to LPS [46, 47], dietary or pharmacological challenges [48–52], and noise insults [53, 54]. In the present study, we used the HMDP to further elucidate the genetic architecture of asthma-related phenotypes in mice and identify loci that modulate AHR in response to diesel exhaust particles (DEP), as a model traffic-related air pollutant.

## Results

### Lung function varies among HMDP strains

**Fig 1:**
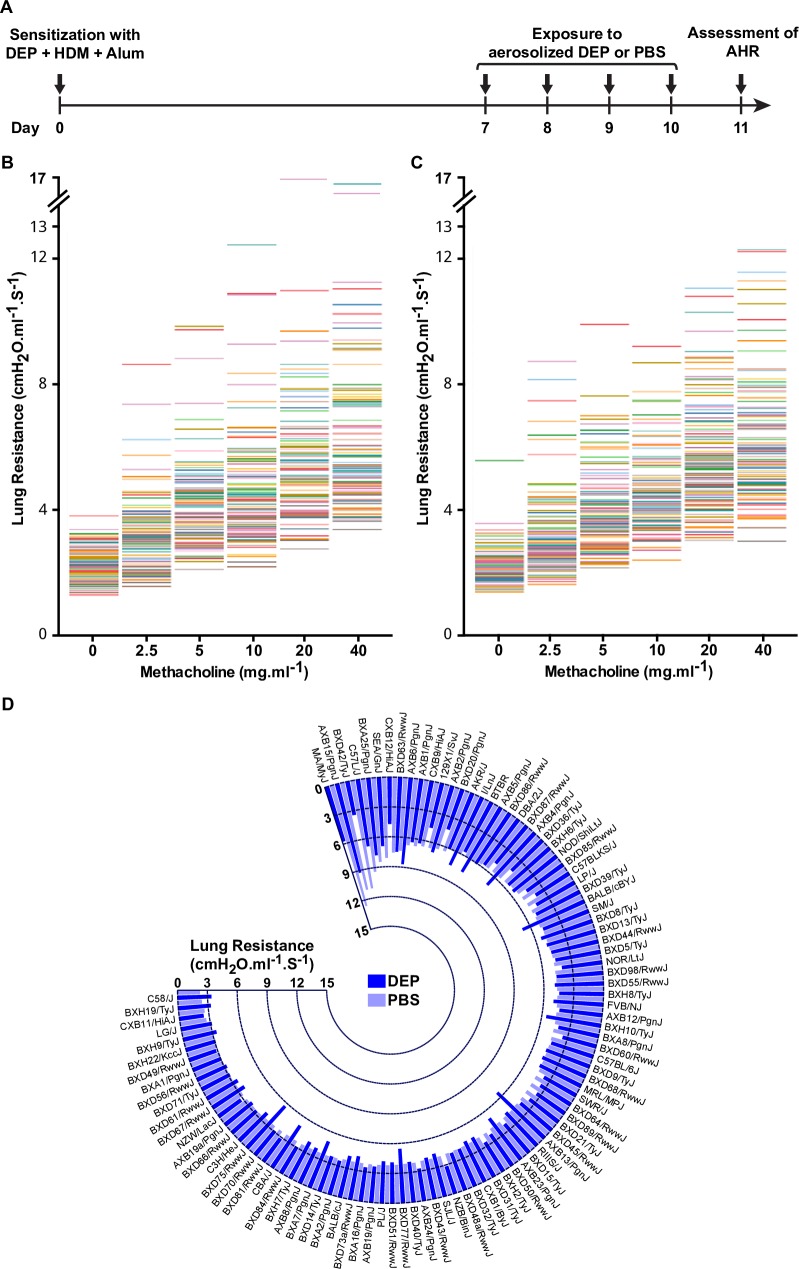
Variation in AHR among 101 inbred strains of HMDP. **A**) Timeline of the sensitization and exposure protocol that was designed to sufficiently induce AHR across 101 HMDP strains of different genetic backgrounds and varying susceptibilities to DEP. Mice (n = 4–8 per strain) were first sensitized on day 0 through a 100μl intraperitoneal injection containing 200μg DEP and 25μg HDM + 2.25mg Alum, as an adjuvant. On days 7–10, mice were separated into two groups and placed in insulated chambers daily for 20mins supplied with ambient air and saturated with either aerosolized PBS (control; n = 2-4/strain) or 200μg DEP (exposed; n = 2-4/strain). To avoid batch effects, the same collection of DEP and lot of HDM was used for all strains and all exposures were carried out at the same time in the mornings. On day 11, airway hyperreactivity was measured by invasive plethysmography. Lung resistance at baseline and in response to increasing concentrations of methacholine is shown for each strain under control PBS exposure (**B**) and DEP-exposed conditions (**C**). Each colored line represents the maximum lung resistance of each strain (as an average of 2–4 mice) at each methacholine dose. **D**) Circular bar-graph shows variation in lung resistance among HMDP mice strains under PBS control (light blue bars) and DEP exposed conditions (dark blue bars) at a methacholine dose of 10mg/ml. Each bar represents the mean value of 2–4 mice per group per strain.

As an initial step towards identifying the genetic factors and GxE interactions underlying asthma-related traits in mice, we determined AHR in 101 strains from the HMDP (**S1 Table**) by measuring lung resistance with and without exposure to DEP. To increase lung function responses, mice were first sensitized on day 0 through an intraperitoneal injection of DEP (200μg), HDM (25μg), + 2.25mg Alum as an adjuvant, followed by daily inhalation exposure on days 7–10 to either aerosolized PBS (n = 2–4 mice/strain), as a control, or DEP (n = 2–4 mice/strain) for 20mins. Lung resistance in response to increasing doses of methacholine was then measured on day 11 using invasive plethysmography (**Fig 1A**).

In the control PBS inhalation exposure group, lung resistance among the 101 HMDP strains exhibited 3-4-fold variation at baseline and as a function of increasing doses of methacholine (**Fig 1B**). Similar variation was also observed among strains after inhalation exposure to aerosolized DEP (**Fig 1C**). A comparison of lung resistance in response to 10mg/ml methacholine for each strain after inhalation exposure to PBS or DEP further illustrates the variation observed among the HMDP (**Fig 1D**). Interestingly, strains that exhibited the highest lung resistance after PBS exposure were not necessarily the same as those with high lung resistance after DEP exposure. For example, at a 10mg/ml dose of methacholine, strain BXD42/TyJ has the third highest lung resistance (11.0 cmH_2_O.ml^-1^.S^-1^) after inhalation exposure to PBS, but exhibits a more subdued response (3.4 cmH_2_O.ml^-1^.S^-1^) after DEP inhalation exposure (**Fig 1D**). Given that the same exposure protocol was used to characterize all strains, these findings indicate that genetic background modulates variation in lung resistance with and without inhalation exposure to DEP.

### Mapping of loci for lung resistance in the HMDP

**Fig 2:**
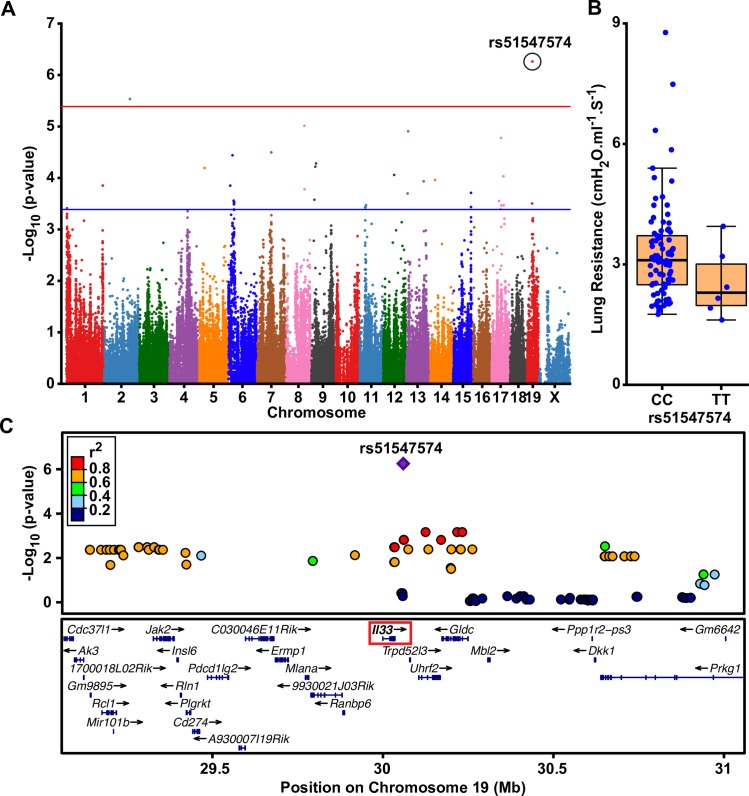
Results of a GWAS for AHR in the HMDP under PBS-control exposure. **A**) The Manhattan plot shows lung resistance under control PBS exposure is significantly associated with two loci on chromosomes 2 and 19. The GWAS analysis included 196,591 SNPs, whose genomic positions are shown along the x-axis with their corresponding -log_10_ p-values indicated by the y-axis. The genome-wide thresholds for significant (p = 4.1x10^-6^) and suggestive (p = 4.1x10^-4^) evidence of association are indicated by the horizontal red and blue lines, respectively. **B**) Lung resistance at a methacholine dose of 2.5mg/ml is lower among strains carrying the minor T allele of the peak SNP (rs51547574) on chromosome 19 compared to strains carrying the C allele. Dots represent the average of 2–4 mice per strain. **C**) Regional plot for the chromosome 19 locus shows that rs51547574 is located directly over *Il33* (red box) although SNPs in strong LD encompass other genes as well.

We next used the phenotype data generated in the HMDP to identify the genetic determinants of AHR under control PBS conditions. A GWAS analysis for AHR at individual methacholine doses identified two loci on chromosomes 2 and 19 that were significantly associated with peak lung resistance in response to 2.5mg/ml of methacholine in PBS-exposed mice (**Fig 2A**). Several suggestively associated loci were also observed on chromosomes 6, 8, 13 and 17 (**Fig 2A**). The lead SNP on chromosome 2 (rs27250829) yielded a p-value of 3.0x10^-6^ and had a minor allele frequency (MAF) of 0.20, whereas the lead SNP on chromosome 19 (rs51547574) was slightly more frequent (MAF = 0.26) and one magnitude more strongly associated with lung resistance (p = 5.6x10^-7^). As shown in **Fig 2B**, the minor T allele of rs51547574, which is also the non-C57BL/6 allele, was associated with lower peak lung resistance in response to 2.5mg/ml methacholine in control PBS-exposed mice. While the most significant association with lung resistance was at a methacholine dose of 2.5mg/ml, a similar pattern of association was observed with varying levels of significance at other doses as well (**Table 1**). Interestingly, association with the chromosome 19 locus was observed at the baseline AHR measurement before administration of methacholine (**Table 1**), suggesting that the effect of this locus on lung resistance manifests even under basal conditions and in the absence of stimulating bronchial constriction. In addition, rs51547574 is located 24kb downstream of *Il33*, although other genes, such as *Trpd52l3*, *Uhrf2*, and *Gldc*, are also located within the interval encompassing other tightly linked SNPs (r^2^≥0.8) (**Fig 2C**). To prioritize among these positional candidates, we next leveraged gene expression data available in the HMDP from several tissues, including liver, adipose, heart, aorta, bone, and macrophages [55]. Of the genes within the high linkage disequilibrium (LD) block on chromosome 19, rs51547574 yielded a *cis* expression quantitative trait locus (eQTL) for *Il33* in heart (p = 4.1x10^-4^) and adipose (p = 0.04). Although not conclusive due to these eQTLs being observed in tissues not directly relevant to asthma or lung function, these results suggest that *Il33* could represent at least one of the candidate causal genes at the chromosome 19 locus.

**Table 1:** Effect Sizes and significance levels at varying doses of methacholine for loci identified for AHR on chromosomes 19 and 3.

				Methacholine Dose (mg.ml^-1^)					
Chromosome	SNP	Exposure	Factor	0	2.5	5	10	20	40
19	rs51547574	PBS	*Beta (C allele)	0.129	0.221	0.097	0.148	0.051	0.036
			**p-value	1.5x10^-5^	**5.6x10**^**-7**^	1.9x10^-2^	3.7x10^-4^	1.9x10^-1^	3.4x10^-1^
3	rs30880385	ΔDEP-PBS	*Beta (T allele)	-0.060	-0.137	-0.040	-0.571	-0.111	-0.045
			**p-value	5.5x10^-1^	2.2x10^-1^	7.3x10^-1^	**2.5x10**^**-6**^	3.3x10^-1^	7.1x10^-1^

*Betas are shown for the indicated effect allele as normal inverse transformed values.

**Genome-wide significant associations are shown in bold.

### GxE GWAS for lung resistance in response to DEP

**Fig 3:**
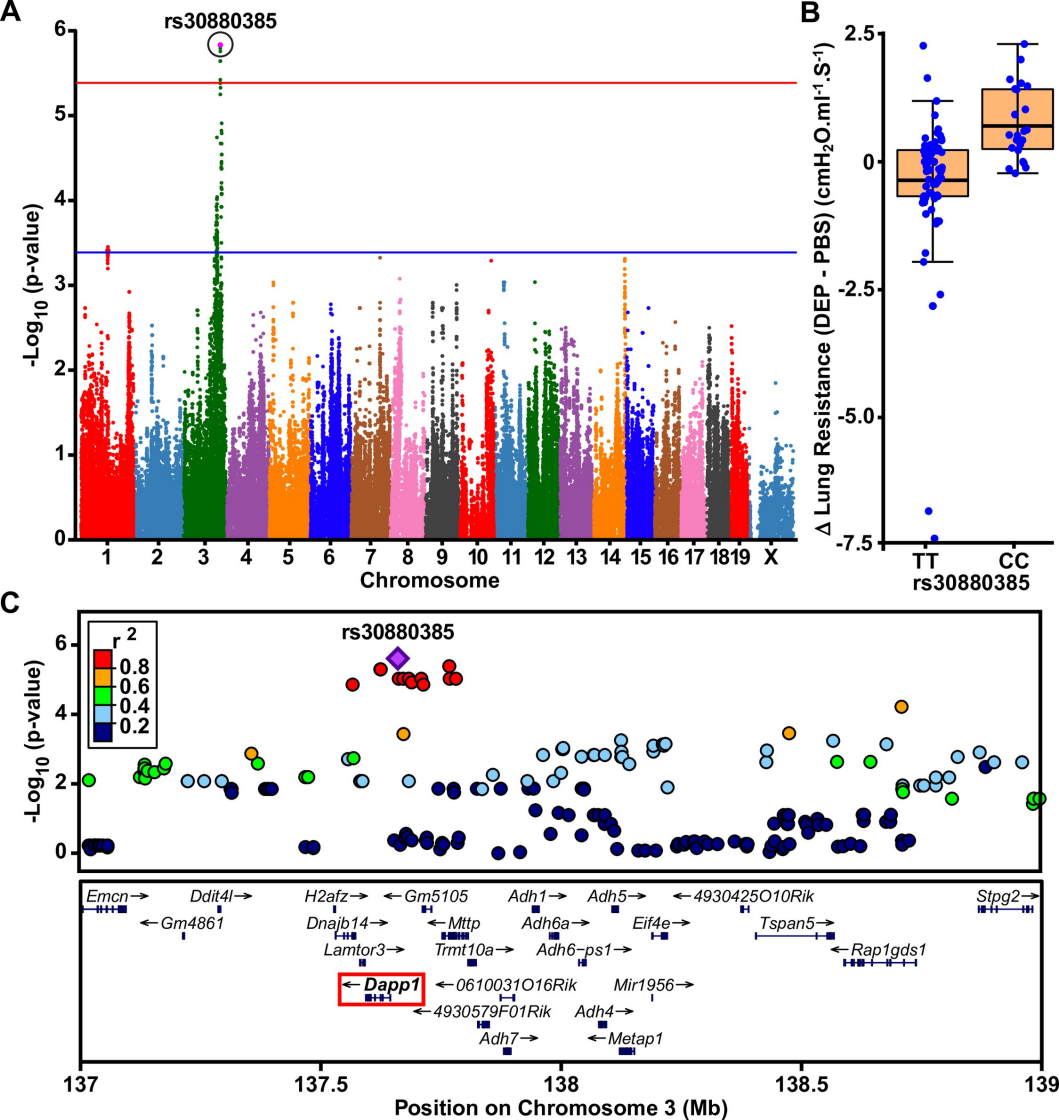
Results of a GxE GWAS for AHR in the HMDP in response to DEP exposure. **A**) The Manhattan plot shows that lung resistance at a methacholine dose of 10mg/ml is significantly associated with a locus on chromosome 3. The GWAS analysis included 203,074 SNPs, whose genomic positions are shown along the x-axis with their corresponding -log_10_ p-values indicated by the y-axis. The mean difference (delta, Δ) in lung resistance between DEP-exposed mice and PBS-exposed controls were calculated for each strain and used in a linear mixed-model GWAS analysis with normal inverse transformed traits as implemented in the program pylmm. Genome-wide thresholds for significant (p = 4.1x10^-6^) and suggestive (p = 4.1x10^-4^) evidence of association are indicated by the horizontal red and blue lines, respectively. **B**) The Δ lung resistance at a methacholine dose of 10mg/ml is greater among strains carrying the minor C allele of the peak SNP (rs30880385) on chromosome 3 compared to strains carrying the T allele. Dots represent mean strain difference in Δ lung resistance between DEP-exposed mice and PBS-exposed controls. **C**) Regional plot for chromosome 3 locus shows that rs30880385 is located directly over *Dapp1* (red box) although SNPs in strong LD encompass other genes as well.

We next applied the same strategy to identify the genetic determinants of lung resistance after inhalation exposure to DEP. For these analyses, we calculated the mean strain difference in peak lung resistance in DEP-induced and control PBS exposures (delta, Δ AHR_DEP_—AHR_PBS_) at each dose of methacholine, as well as Δ area under the curve (AUC) values across all methacholine doses. A GxE GWAS for AUC-based Δ AHR_DEP_—AHR_PBS_ only identified two suggestively associated loci on chromosomes 4 and 15 (**S1 Fig**). By comparison, analysis of the Δ AHR_DEP_—AHR_PBS_ data at individual methacholine doses revealed a significantly associated locus for lung resistance on chromosome 3 at a methacholine dose of 10mg/ml (lead SNP rs30880385; p = 2.5 x 10^−6^; **Fig 3A**). The minor C allele of rs30880385 is the non-C57BL/6 allele and has a frequency of 0.36 among the HMDP strains. Although there were no genome-wide significant differences in lung resistance under PBS or DEP exposures conditions as a function of rs30880385 genotype (**S2A Fig**), the C allele was associated with a significantly (p = 2.5 x 10^−6^) greater difference in Δ (DEP-PBS) lung resistance at a methacholine dose of 10mg/ml (**Fig 3B** and **S2B Fig**). Analysis of AHR across all methacholine doses using AUC values also revealed a modestly significant difference (p = 0.05) as a function of rs30880385 genotype (**S2C Fig**). Similarly, there was only nominal evidence (p<0.05) for association of this locus with AHR in response to PBS exposure at a methacholine dose of 10mg/ml (**S2 Table**). These observations suggest that association of the chromosome 3 locus with lung resistance is mediated through an interaction with DEP exposure. A regional plot of this locus shows that rs30880385 is located 19kb upstream of *Dapp1* (**Fig 3C**), although other genes located within the region encompassed by tightly linked SNPs (r^2^≥0.8) include *Gm5105*, *Mttp*, and *Lamtor3* (**Fig 3C**). Interrogation of the HDMP gene expression database however did not identify any *cis* eQTLs for *Dapp1*, *Gm5105*, *Mttp*, or *Lamtor3* among the available tissues.

### *DAPP1* is expressed in the lungs and associated with inflammatory cytokines in humans

**Fig 4:**
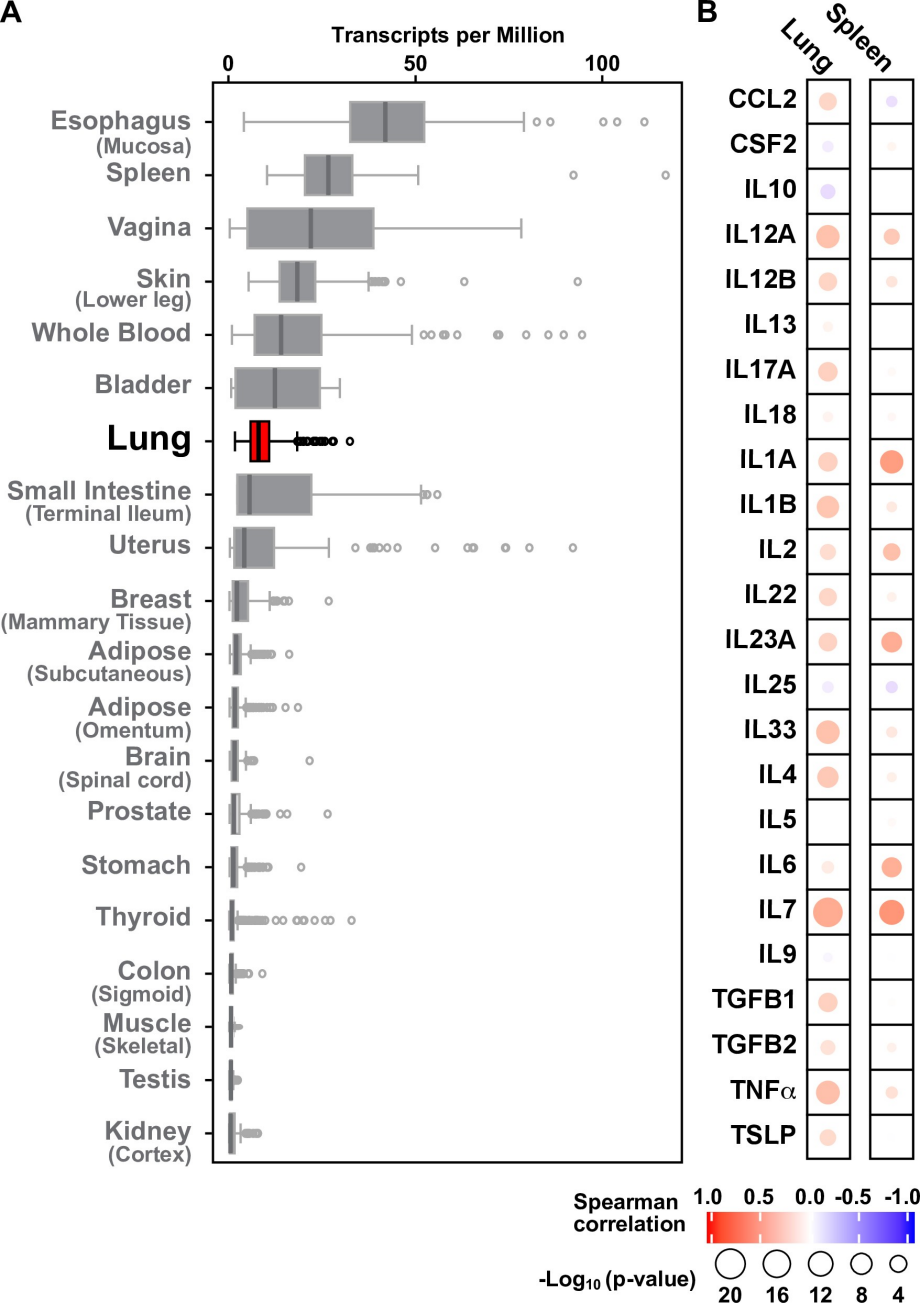
Tissue expression pattern of *DAPP1* and relationship to inflammatory genes in humans. **A**) Levels of *DAPP1* mRNA, expressed as transcripts per million reads (TPM), are highest in mucosal and immune system-related tissues, including lung (shown in red). Data were obtained from the Genotype-Tissue Expression (GTEx) project portal. **B)** *DAPP1* expression in lung and spleen is strongly and positively correlated with several pro-inflammatory cytokines relevant to asthma, including *IL1A*, *IL7*, *IL12A*, *IL17A*, *IL23A*, and *IL33*. The color-range in the illustration reflects the strength of the Spearman correlation coefficients (r) and the size of the circles reflects the significance levels of the p-values.

Although none of the candidate causal genes at the chromosome 3 locus are known to have a biological role in lung function or asthma, previous studies have shown that *Dapp1* encodes a multi-functional adaptor protein involved in T cell activation that is also highly expressed in lymphocytes and lung pneumocytes in mice and humans [56–58]. Since gene expression data available in the HDMP may not have necessarily been in tissues most relevant to the immune system-related functions of *Dapp1*, we next leveraged publicly available mutli-tissue RNAseq data in humans from the GTEx Project [59]. *DAPP1* expression in humans was highest in lymphocytes, spleen, whole blood, and several mucosal tissues, including the lung (**Fig 4A**). Moreover, *DAPP1* expression in the lung was highly positively correlated with a panel of pro-inflammatory cytokines, including several that have been implicated in asthma, such as *IL1A*, *IL7*, *IL12A*, *IL17A*, *IL23A*, and *IL33* (**Fig 4B**). In spleen, *DAPP1* expression was similarly positively associated with expression of cytokines, but the pattern was characterized by fewer and less significant correlations than those in the lung (**Fig 4B**). A similar analysis in the HMDP with *Dapp1* did not reveal significant correlations with expression of the same pro-inflammatory cytokine genes among the available tissues. Nonetheless, based on the multiple significant correlations observed in humans, we reasoned that *Dapp1* could be at least one of the genes at the chromosome 3 locus that mediated the effects of DEP on lung resistance in mice.

### Functional validation of *Dapp1* as an exposure-responsive gene

**Fig 5:**
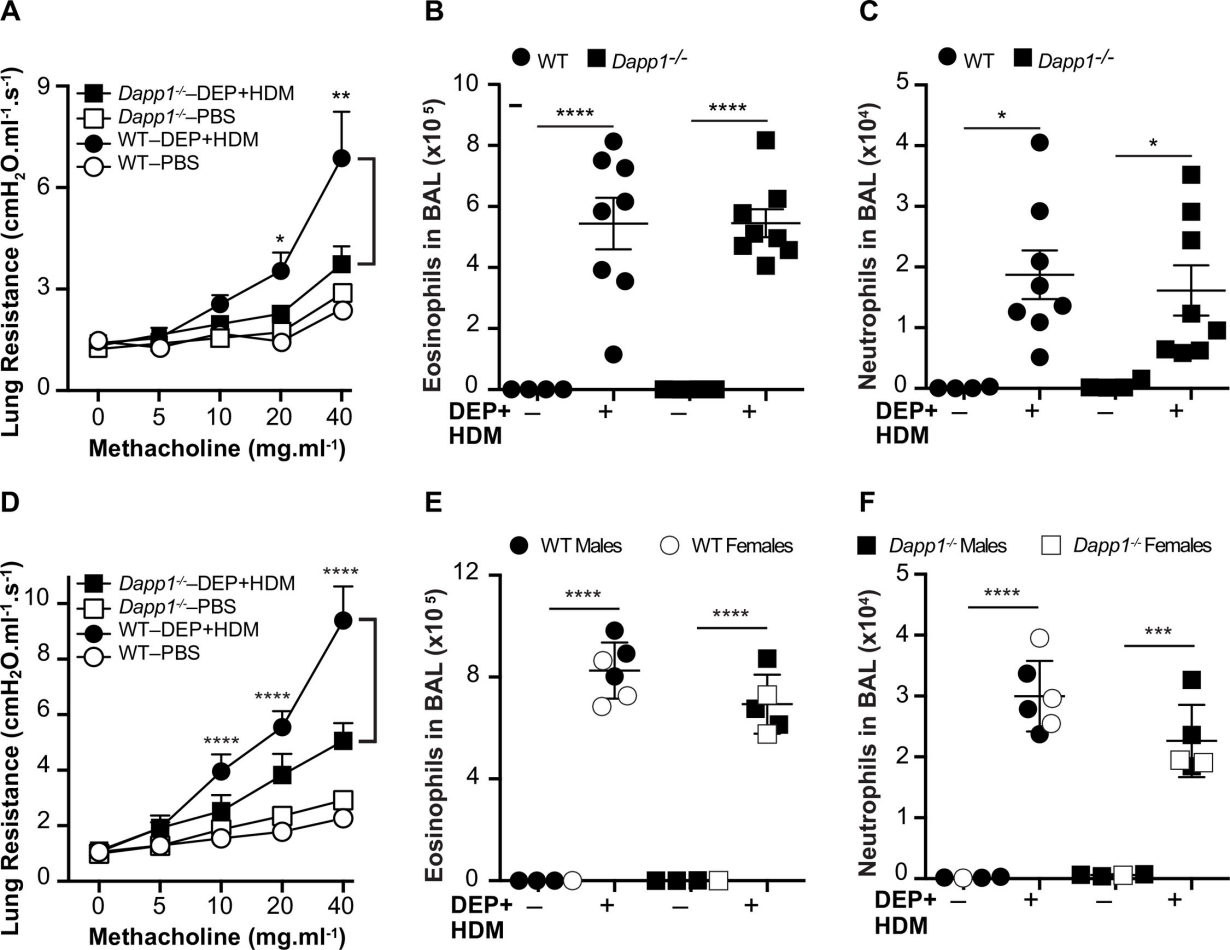
Functional validation of *Dapp1* as a DEP-responsive gene. **A** and **D)** Lung resistance after inhalation exposure to DEP and HDM is not increased in *Dapp1*^*-/-*^ mice compared to WT animals. DEP/HDM exposure induced eosinophilia **(B** and **E)** and neutrophilia **(C** and **F)** to the same extent in BAL fluid from *Dapp1*^*-/-*^ and WT mice. Mice were first sensitized on day 0 through a 100μl intraperitoneal injection containing 200μg DEP and 25μg HDM + 2.25mg Alum, as an adjuvant. On days 7–10, mice were placed in insulated chambers daily for 20mins supplied with ambient air and saturated with either aerosolized PBS or both 200μg DEP and 25μg HDM. On day 11, airway hyperreactivity was measured by invasive plethysmography, followed by collection of BAL. Cell counts in BAL fluid were determined by flow cytometry. WT control animals were either female C57BL/6J mice purchased from the Jackson Laboratories (**A-C**) or WT littermates of both sexes generated through an intercross between *Dapp1*^+*/-*^ heterozygote mice (**D-F**). For both experiments, n = 4–8 mice in each group. Data are shown as mean ± SE. ****p<0.0001; ***p<0.001; **p<0.005; *p<0.05.

We next sought to functionally validate the GxE association signal on chromosome 3 using an *in vivo* approach with *Dapp1* deficient (*Dapp1*^*-/-*^) mice. We first used the same exposure protocol as for the HMDP strain survey (**Fig 1A**) to characterize female *Dapp1*^*-/-*^ mice and female wildtype (WT) C57BL/6J controls purchased from the Jackson Laboratories. As shown in **S3 Fig**, there was a non-significant trend for a difference between DEP-exposed *Dapp1*^*-/-*^ mice and WT controls. Therefore, we repeated these experiments a second time using the same dual exposure sensitization step with DEP and HDM. However, mice underwent inhalation exposure to both DEP and HDM on days 7–10, followed by measurement of AHR on day 11. The rationale for this strategy was that the combination of dual exposures with DEP and HDM for both sensitization and inhalation would induce lung resistance sufficiently to reveal a phenotypic difference between *Dapp1*^*-/-*^ mice and WT mice. In addition, we carried out two independent experiments using female WT C57BL/6J controls purchased from either the Jackson Laboratories or male and female WT littermates generated through an intercross between *Dapp1*^*+/-*^ mice. In both experiments, inhalation exposure to DEP and HDM robustly induced AHR in control mice and WT littermates (**Fig 5A** and **5D**). However, the induction of lung resistance by DEP was significantly blunted in *Dapp1*^*-/-*^ mice, particularly at methacholine doses of 20 and 40mg/ml (**Fig 5A** and **5D**). These differences were observed in experiments using only female mice as well as in those where mice of both sexes were used. By comparison there were no differences between WT or *Dapp1*^*-/-*^ mice under control PBS conditions (**Fig 5A** and **5D**). To examine whether reduced lung resistance in response to DEP in *Dapp1*^*-/-*^ was associated with modulation of airway inflammation, we characterized the cellular composition of bronchoalveolar lavages (BAL) from *Dapp1*^*-/-*^ and WT mice. Inhalation exposure to DEP/HDM significantly increased eosinophil and neutrophil numbers in the BAL of both *Dapp1*^*-/-*^ animals and control mice/WT littermates compared to the corresponding PBS-exposed control groups (**Fig 5B, 5C, 5E and 5F**). However, there were no significant differences in eosinophil and neutrophil counts in the BAL of *Dapp1*^*-/-*^ mice and either set of WT controls littermates after inhalation exposure to DEP/HDM (**Fig 5B, 5C, 5E and 5F**). Taken together, these data corroborate the GxE GWAS results on chromosome 3 and provide functional evidence that *Dapp1* modulates lung resistance in response to DEP through mechanisms that are independent of eosinophil and neutrophil recruitment to the lung.

## Discussion

In the present study, we characterized asthma-related traits in over 100 inbred strains of mice from the HMDP to facilitate identification of genetic determinants for AHR, particularly after exposure to a model traffic-related air pollutant. These efforts demonstrated that lung function across the HMDP varied several fold under control exposures as well as in response to DEP and revealed several significantly and suggestively associated loci for AHR. At one locus in particular, we functionally demonstrated that *Dapp1* modulates lung resistance in response to DEP exposure, thus providing evidence for a novel GxE interaction that influences asthma-related traits in mice. Taken together, these findings represent one experimental approach to understanding the genetic factors that determine susceptibility to asthma in response to environmental pollutants that are known to have large independent adverse effects on lung function/growth, especially in children [60–62].

Two important considerations in our studies are whether the identified loci corroborate results of previous mouse studies and whether the identified genetic pathways are relevant to humans. In this regard, we mapped lung resistance under control conditions to two loci on chromosome 2 and 19. Interestingly, our lead SNP (rs27250829) on chromosome 2 maps to position 137,425,632 (based on build NCBI37/mm9), which is within the ~36Mb interval (chr 2:122,652,343–163020182) spanning a QTL for AHR previously reported in mice [63]. By comparison, of the genes within the relatively small LD block on chromosome 19, a *cis* eQTL were only observed for *Il33*, providing functional evidence that this cytokine gene is at least one candidate causal gene at this locus. Although these results are not entirely conclusive since the eQTLs for were only modestly significant and observed in tissues not directly relevant to asthma, it is noteworthy that syntenic association signals for asthma have been identified with rare and common genetic variation at the *IL33* locus in multiple human studies [15–34, 64]. These observations support the notion that some of the genetic determinants for asthma-related phenotypes may be shared between mice and humans.

The GxE locus we identified on chromosome 3 in response to DEP contained several genes without an obvious role in lung function or asthma-related traits. Of these, we prioritized *Dapp1* because of its known role in mediating activation of and interactions between immune cells [65–69], its expression profile in tissues relevant to asthma, and the high correlation of its mRNA levels with those of several asthma-related inflammatory cytokines in humans. In two independent experiments with genetically targeted mice, we functionally validated *Dapp1* as an exposure-responsive gene at the chromosome 3 locus. It should be noted that a phenotypic difference between *Dapp1*^*-/-*^ and WT mice was observed after inhalation exposure to both DEP and HDM, which is slightly different from the protocol we used for the HMDP strain survey. Thus, when on the background of a relatively resistant strain (C57BL/6J), it is possible that both triggers are required in order for the protective effect of *Dapp1* deficiency on induction of AHR to manifest. Regardless, a mechanism(s) for how *Dapp1* would be involved in lung resistance, particularly in response to DEP/HDM, is still not directly evident. It is also not known whether Dapp1 mediates a response to other exposures as well. Interestingly, *DAPP1* expression has been shown to change in human bronchial epithelial cells after exposure to cigarette smoke [70]. Taken together with our results, these observations suggest that *Dapp1* potentially mediates exposure-induced lung resistance through immune system-related mechanisms. This notion would be consistent with the apparent strong immunological component that GWAS studies in humans have indicated for asthma based on the positional candidate genes located at the identified loci [31]. However, a large human GWAS did not reveal any main effect associations between the *DAPP1* locus and asthma [35], including with SNPs that yielded strong multi-tissue *cis* eQTLs for *DAPP1* and other genes in the syntenic region [59]. Additional studies will be needed in order to obtain a more definitive understanding of how *Dapp1* function influences AHR and its relevance to the biological mechanisms and GxE interactions of asthma in humans.

Compared to humans, inbred strains provide certain advantages for GxE studies, including the ability to tightly control environmental exposures across strains and the ability to phenotype multiple individuals of the same genotype. While we used the HMDP to search for genetic determinants of AHR after DEP exposure, analogous strategies with various other mouse genetics platforms have also been used to explore GxE interactions underlying allergen-induced asthma traits [39]. For example, Kelada et al. used the Collaborative Cross [71, 72] to identify loci for allergic airway disease with and without exposure to HDM that were distinct from those identified herein. Collectively, these findings illustrate the utility of leveraging natural genetic variation with inbred mouse strains to identify both main genetic effects and GxE interactions for asthma-related phenotypes, as well as the existence of exposure-specific GxE loci.

While the present analyses have revealed novel genetic determinants of AHR in mice, our study should also be taken in the context of certain limitations. First, our exposure protocol was relatively short-term compared to the chronic nature of how humans are typically exposed to environmental pollutants. Second, we focused on DEP as a model traffic-related air pollutant and sensitized mice with HDM in conjunction with Alum as an adjuvant. We chose this strategy based on the notion that immune responses in humans are potentiated by natural occurring adjuvants and prior mouse studies where it has been shown that Alum promotes the development of lung resistance in combination with asthma triggers, such as DEP [73]. However, it is possible that other components of DEP could be causing subsequent immune responses by virtue of being in the immunization mix with Alum or that DEP could exacerbate already existing allergic airway disease. Alternatively, it is not known whether the GxE interactions we identified herein are specific to DEP or apply to other exposures as well. Addressing these possibilities will require appropriately designed studies. Lastly, the ~100 HMDP inbred strains, which originate from a small number of founders, provide limited genetic diversity and relatively lower power to detect loci compared to large outbred human populations. This may explain, at least in part, why we only mapped a small number of loci for lung resistance in the HMDP whereas human GWAS have identified ~200 genomic regions for asthma-related traits.

In conclusion, we identified several loci associated with AHR in mice, including *Dapp1* as a determinant of lung resistance in response to DEP exposure. These results may have implications for genetic susceptibility to asthma in humans, which will need to be addressed using cohorts in which traffic-related air pollution exposure estimates are available along with genotype and phenotype information. Moreover, these results demonstrate the utility of the HMDP for dissecting the genetic architecture of complex traits, particularly for those phenotypes that are strongly influenced by environmental exposures.

## Methods

### Ethics statement

All animal studies were approved by the USC Keck School of Medicine Institutional Animal Care and Use Committee and conducted in accordance with the Department of Animal Resources’ guidelines. Human studies were conducted with publicly available de-identified gene expression data from the GTEx Project [59].

### Mice and HMDP panel

Six to eight-week-old female mice of 101 strains (n = 4–8) were purchased from the Jackson Laboratories. A full list of strain names and stock numbers are provided in the **S1 Table**. Biological validation of *Dapp1* was carried out using previously generated genetically modified mice bred onto a C57Bl/6J background that were kindly provided by Dr. Aaron Marshall [68]. Control animals for the first set of exposure experiments with female *Dapp1*^*-/-*^ mice were female C57BL/6J mice purchased from the Jackson Laboratories. For the second set of exposure experiments, *Dapp1*^*-/-*^ and WT littermates of both sexes were generated through an intercross between *Dapp1*^+*/-*^ heterozygotes. All mice in the study, including the *Dapp1*^*-/-*^ and 101 HMDP strains, were housed and bred in the same vivarium room. Mice were maintained on a 12-hour light/dark cycle in sterilized microisolator cages and received autoclaved food and water ad libitum.

### Exposure protocol and measurement of AHR

DEP (engine combustion products) were collected from a light-duty, four-cylinder diesel engine (type 4JB1, Isuzu, Japan) under a 10-torque load in a cyclone impactor using standard diesel fuel, as described previously [74]. The collected DEP were then suspended in PBS as one batch with multiple aliquots that were used for intraperitoneal injections directly or aerosolized for inhalation exposures using Aeroneb ultrasonic nebulizers (Aerogen, Chicago, IL) with a pore size of 4–5μM. Based on prior mouse [75, 76] and human [77, 78] studies and our own pilot experiments with two relatively sensitive (Balb/cByJ) and resistant (C57BL/6J) mouse strains, we designed an exposure protocol that could be uniformly adapted across ~100 strains of different genetic backgrounds and varying susceptibilities to DEP (additional details are provided in the **S1 Appendix**). To sufficiently induce AHR, mice from the HMDP (n = 4–8 per strain) were first sensitized on day 0 through a 100μl intraperitoneal injection containing 200μg DEP and 25μg HDM (Stallergenes Greer, Lenoir, NC) + 2.25mg Alum as an adjuvant [73]. On days 7–10, mice were separated into two groups and placed in insulated chambers daily for 20mins supplied with ambient air and saturated with aerosolized PBS (control; n = 2-4/strain) or 200μg DEP (exposed; n = 2-4/strain). To avoid batch effects, the same collection of DEP and lot (# 145793) of HDM was used for all strains and all exposures were carried out at the same time in the mornings. Based on prior analyses [79], there was minimal (0.01%) contamination of the HDM extract we used with endotoxin (0.1μg LPS/mg HDM). On day 11, AHR was measured by invasive plethysmography. Lung resistance and dynamic compliance at baseline and in response to sequentially increasing doses of methacholine was measured using a Buxco FinePointe (Data Sciences International, St. Paul, MN) respiratory system in tracheostomized immobilized mice that were mechanically ventilated under general anesthesia, as described elsewhere [80].

### Characterization of *Dapp1*^*-/-*^ mice

For exposure experiments with *Dapp1*^*-/-*^ mice and WT controls or littermates, mice were first sensitized on day 0 with 200μg DEP and 25μg HDM (Stallergenes Greer, Lenoir, NC) + 2.25mg Alum. This was followed by one set of inhalation experiments that mimicked the exposure paradigm used for the HMDP where mice were exposed daily to aerosolized PBS or 200μg DEP for 20mins on days 7–10, followed by measurement of AHR on day 11. To induce lung resistance further, a second set of inhalation experiments were also conducted where mice were first sensitized in the same manner with 200μg DEP and 25μg HDM but underwent daily inhalation exposure to aerosolized PBS or both 200μg DEP and 25μg HDM for 20mins on days 7–10, followed by measurement of AHR on day 11. For both exposure studies, BAL fluid was collected after completion of AHR measurements by cannulating the trachea and washing the airways three times with 1mL PBS. The fluid was recovered each time and centrifuged at 400g for 7mins to pellet the cells. Leukocytes in BAL fluid were quantified by flow cytometry after staining with phycoerythrin (PE)-anti-Siglec-F (BD Biosciences, San Jose, Calif), fluorescein isothiocyanate (FITC)-anti-CD19, peridinin-chlorophyll-protein complex (PerCP)/Cy5.5-anti-CD3ε, allophycocyanin (APC)-anti-Gr-1, PE/Cy7-anti-CD45, APC/Cy7-anti-CD11c (BioLegend, San Diego, Calif), and eFluor450-anti-CD11b (eBioscience, San Diego, Calif) in the presence of anti-mouse FC-block (BioXcell, West Lebanon, NH). CountBright absolute count beads (Thermo Fisher Scientific, Waltham, Mass) were used to calculate absolute cell number, according to the manufacturer’s instructions. At least 10^5^ CD45^+^ cells were acquired on a BD FACSCanto II (BD Biosciences). Data were analyzed with FlowJo software (TreeStar, Ashland, OR).

### GWAS analyses for main effects and GxE interactions

GWAS analyses in the HMDP were carried out for main SNP effects under control PBS conditions at the individual mouse level with lung resistance measurements obtained at each methacholine dose. For the GxE interaction analyses, we derived DEP-induced AHR traits by calculating the mean strain difference (delta, Δ) in lung resistance at each dose of methacholine between DEP-exposed mice and PBS-exposed controls. These calculated Δ values were then analyzed at each methacholine dose. In addition, we also tested for association with AHR data that incorporated lung resistance measurements at all methacholine doses. This was done by calculating mean strain differences in area under the curve (AUC) values between DEP-exposed and PBS-exposed mice (Δ AUC_DEP_—AUC_PBS_). Given this analytical approach, the statistical model used to test for GxE interactions did not include terms for the main effects of SNPs and DEP exposure. Genotypes for ~450,000 SNPs in the HMDP strains were obtained from the Mouse Diversity Array [81]. SNPs were required to have minor allele frequencies >5% and missing genotype frequencies <10%. Applying these filtering criteria resulted in a final set of ~200,000 SNPs that were used for analysis. Association testing was performed using python-based linear mixed models with log or normal inverse transformed traits as implemented in the program pylmm [82], which has the advantage of accounting for population structure among the inbred strains in the HMDP. Genome-wide significance threshold in the HMDP was determined by the family-wise error rate as the probability of observing one or more false positives across all SNPs per phenotype. We ran 100 different sets of permutation tests and parametric bootstrapping of size 1,000 and observed that the genome-wide significance threshold at a family-wise error rate of 0.05 corresponded to a p-value of 4.1x10^-6^, similar to what we have used in previous HMDP studies [41–45]. This is approximately one order of magnitude larger than the threshold obtained by Bonferroni correction (4.6x10^-7^), which would be an overly conservative estimate of significance since nearby SNPs among inbred mouse strains are highly correlated with each other.

### Bioinformatics analyses with *DAPP1* in humans

Gene expression data for *DAPP1* in various tissues and asthma-related pro-inflammatory cytokine genes in lung and spleen were taken from the GTEx Project [59]. The relationship between *DAPP1* and pro-inflammatory cytokines genes was tested by Spearman’s correlation analyses. Data were analyzed using R [83] and correlations were considered significant at a p-value<0.05.

### Statistical analyses

Differences in lung resistance between *Dapp1*^*-/-*^ mice and WT controls were analyzed by two-way ANOVA and generalized linear models with log transformed data, followed by a Tukey post-hoc test. Cell counts in the BAL from PBS control and DEP-exposed WT and *Dapp1*^*-/-*^ mice were compared using a Kruskal–Wallis test followed by Dunn's multiple comparisons non-parametric test. Data were analyzed using GraphPad Prism v6.04 and differences were considered significant at a p-value<0.05.

## Supporting information

S1 Appendix Additional details and considerations for the exposure protocol. (DOCX)

S1 Table List of HMDP strains used in the study. (DOCX)

S2 Table Association of lead SNP (rs30880385) at GxE locus on chromosome 3 with lung resistance under control PBS conditions. (DOCX)

S1 Fig Results of a GWAS for AHR in the HMDP in response to DEP exposure. The Manhattan plot shows the results of a GWAS analysis for lung resistance after inhalation exposure to DEP across all methacholine doses, as determined by (AUC) analysis. No locus was genome-wide significant but two loci on chromosomes 4 and 15 exhibited suggestive evidence for association. To be consistent with the phenotype that led to the identification of the chromosome 3 GxE locus, differences (delta, Δ) in AUC values between DEP and PBS (Δ AUC_DEP_—AUC_PBS_) were used for this analysis. The GWAS included 203,074 SNPs, whose genomic positions are shown along the x-axis with their corresponding -log10 p-values indicated by the y-axis. The genome-wide thresholds for significant (p = 4.1x10^-6^) and suggestive (p = 4.1x10^-4^) evidence of association are indicated by the horizontal red and blue lines, respectively.
(TIFF)

S2 Fig Genotypic effect of lead SNP at chromosome 3 GxE Locus on lung resistance among HDMP strains. **A)** Lung resistance is plotted as a function of exposure (PBS or DEP) group and genotypes at rs30880385 across increasing doses of methacholine. **B)** The difference (delta, Δ) in lung resistance between DEP exposure and PBS control groups is plotted as a function of genotype across increasing doses of methacholine. The Δ value at 10mg/ml methacholine was significantly different (p = 2.5x10^-6^) between strains with CC (n = 25) and TT (n = 72) genotypes at rs30880385 and the basis for identification of the chromosome 3 locus in the GxE GWAS. **C)** The difference in lung resistance between DEP exposure and PBS control groups across all methacholine doses, as calculated by an area under the curve (Δ AUC_DEP_—AUC_PBS_), was also significantly different as a function of genotype. Data are shown as mean ± SE. ****p<0.0001; *p<0.05.
(TIFF)

S3 Fig Evaluation of *Dapp1* as a DEP-responsive gene. **A)** Lung resistance after inhalation exposure to DEP is not statistically significantly different between female *Dapp1*^*-/-*^ mice compared to control female WT mice but shows a trend for being decreased. There were also no differences in the induction of eosinophilia **(B)** and neutrophilia **(C)** in BAL fluid from *Dapp1*^*-/-*^ and control WT mice after inhalation exposure to DEP. Mice (n = 4–6 per strain) were first sensitized on day 0 through a 100μl intraperitoneal injection containing 200μg DEP and 25μg HDM + 2.25mg Alum as an adjuvant. On days 7–10, mice were placed in insulated chambers daily for 20mins supplied with ambient air and saturated with aerosolized PBS (as a control) or 200μg DEP, followed measurement of AHR by invasive plethysmography and collection of BAL fluid on day 11. WT control animals were C57BL/6J mice purchased from the Jackson Laboratories. Cell counts in BAL fluid were determined by flow cytometry. Data are shown as mean ± SE.
(TIFF)
