# Supplementary material for: A GWAS approach identifies Dapp1 as a determinant of air pollution-induced airway hyperreactivity
Source: PLoS Genet. 2019 Dec 23;15(12):e1008528. doi: 10.1371/journal.pgen.1008528 (PMC6944376; doi:10.1371/journal.pgen.1008528)
Supplement: S1 Appendix — (DOCX) [file pgen.1008528.s001.docx]

**Exposure Protocol:** We designed the mouse exposure experiments based on the generally accepted premise that development of asthma in humans is a complex process involving genetic predisposition and exposure to triggers in the presence of naturally occurring adjuvants, e.g. viruses. It is thought that these adjuvants potentiate immune responses through upregulation of pattern recognition receptors, such as toll-like receptors, which ultimately upregulate the inflammasome and induce airway inflammation [1, 2]. Based on these observations, Alum has been used as an adjuvant in mouse models of asthma where it has also been shown to promote the development of lung resistance in combination with asthma triggers, such as DEP or HDM [3]. In addition to these considerations, we also sought to develop an exposure protocol that could be uniformly adopted across ~100 mouse strains of different genetic backgrounds and varying susceptibilities to DEP. For example, it would be important to induce AHR sufficiently in strains that are relatively resistant to DEP exposure but not to the extent that it would overwhelm more sensitive strains. Since AHR data in response DEP exposure among ~100 strains were not available to us *a priori*, we used data from previous studies [4, 5] to carry out a series of pilot experiments with two relatively sensitive (Balb/cByJ) and resistant (C57BL/6J) strains. In the first set of experiments, mice were sensitized with only DEP, followed by inhalation exposure to DEP or PBS on 4 consecutive days one week later. This approach did induce AHR but potentially not enough to generate sufficient variation across ~100 mouse strains. Therefore, we carried out a second series of pilot experiments where mice were sensitized with *both* DEP and HDM in order to further potentiate the induction of AHR, which was again followed by the inhalation exposures with DEP alone or PBS. Based on the results of these two pilots, we concluded that the optimal exposure strategy for the HMDP strain survey would be to first mice sensitize with both DEP (200μg) and HDM (25μg) in the presence of Alum (2.25mg) as an adjuvant, followed by serial exposures to DEP one week afterwards.

**Supplemental References:**

1. Hirota JA, Knight DA. Human airway epithelial cell innate immunity: relevance to asthma. Curr Opin Immunol. 2012;24(6):740-6. doi: 10.1016/j.coi.2012.08.012. PubMed PMID: 23089231.

2. Brusselle GG, Provoost S, Bracke KR, Kuchmiy A, Lamkanfi M. Inflammasomes in respiratory disease: from bench to bedside. Chest. 2014;145(5):1121-33. doi: 10.1378/chest.13-1885. PubMed PMID: 24798836.

3. Kool M, Soullie T, van Nimwegen M, Willart MA, Muskens F, Jung S, et al. Alum adjuvant boosts adaptive immunity by inducing uric acid and activating inflammatory dendritic cells. J Exp Med. 2008;205(4):869-82. doi: 10.1084/jem.20071087. PubMed PMID: 18362170; PubMed Central PMCID: PMCPMC2292225.

4. Acciani TH, Brandt EB, Khurana Hershey GK, Le Cras TD. Diesel exhaust particle exposure increases severity of allergic asthma in young mice. Clin Exp Allergy. 2013;43(12):1406-18. doi: 10.1111/cea.12200. PubMed PMID: 24112543.

5. Brandt EB, Biagini Myers JM, Acciani TH, Ryan PH, Sivaprasad U, Ruff B, et al. Exposure to allergen and diesel exhaust particles potentiates secondary allergen-specific memory responses, promoting asthma susceptibility. J Allergy Clin Immunol. 2015;136(2):295-303 e7. doi: 10.1016/j.jaci.2014.11.043. PubMed PMID: 25748065; PubMed Central PMCID: PMCPMC4530081.
