# Supplementary material for: A GWAS approach identifies Dapp1 as a determinant of air pollution-induced airway hyperreactivity
Source: PLoS Genet. 2019 Dec 23;15(12):e1008528. doi: 10.1371/journal.pgen.1008528 (PMC6944376; doi:10.1371/journal.pgen.1008528)
Supplement: S1 Table — (DOCX) [file pgen.1008528.s002.docx]

**S1 Table. List of HMDP strains used in the study.**

| Strain | Stock # | Strain | Stock # | Strain | Stock # |
| --- | --- | --- | --- | --- | --- |
| 129X1/SvJ | 000691 | BXD44/RwwJ | 007094 | C57BLKS/J | 000662 |
| AKR/J | 000648 | BXD45/RwwJ | 007096 | C57L/J | 000668 |
| AXB1/PgnJ | 001673 | BXD48/RwwJ | 007097 | C58/J | 000669 |
| AXB12/PgnJ | 001683 | BXD49/RwwJ | 007098 | CBA/J | 000656 |
| AXB13/PgnJ | 001826 | BXD5/TyJ | 000037 | CXB1/ByJ | 000351 |
| AXB15/PgnJ | 001685 | BXD50/RwwJ | 007099 | CXB11/HiAJ | 001632 |
| AXB19/PgnJ | 001687 | BXD51/RwwJ | 007100 | CXB12/HiAJ | 001633 |
| AXB19a/PgnJ | 001686 | BXD55/RwwJ | 007103 | CXB9/HiAJ | 001630 |
| AXB2/PgnJ | 001674 | BXD56/RwwJ | 007104 | DBA/2J | 000671 |
| AXB23/PgnJ | 001690 | BXD60/RwwJ | 007105 | FVB/NJ | 001800 |
| AXB24/PgnJ | 001691 | BXD61/RwwJ | 007106 | I/LnJ | 000674 |
| AXB4/PgnJ | 001676 | BXD64/RwwJ | 007109 | LG/J | 000675 |
| AXB5/PgnJ | 001677 | BXD66/RwwJ | 007111 | LP/J | 000676 |
| AXB6/PgnJ | 001678 | BXD67/RwwJ | 007112 | MA/MyJ | 000677 |
| AXB8/PgnJ | 001679 | BXD68/RwwJ | 007113 | MRL/MPJ | 000486 |
| BALB/cByJ | 001800 | BXD70/RwwJ | 007115 | NOD/ShiLtJ | 001976 |
| BALB/cJ | 000651 | BXD71/RwwJ | 007116 | NOR/LtJ | 002050 |
| BTBR_T_tf/J | 002282 | BXD73/RwwJ | 007117 | NZB/BinJ | 000684 |
| BXA1/PgnJ | 001692 | BXD75/RwwJ | 007119 | NZW/LacJ | 001058 |
| BXA11/PgnJ | 001699 | BXD77/RwwJ | 007121 | PL/J | 000680 |
| BXA12/PgnJ | 001700 | BXD8/TyJ | 000084 | RIIIS/J | 000683 |
| BXA16/PgnJ | 001703 | BXD81/RwwJ | 007125 | SEA/GnJ | 000644 |
| BXA2/PgnJ | 001693 | BXD84/RwwJ | 007127 | SJL/J | 000686 |
| BXA25/PgnJ | 001711 | BXD85/RwwJ | 007128 | SM/J | 000687 |
| BXA7/PgnJ | 001696 | BXD86/RwwJ | 007129 | SWR/J | 000689 |
| BXA8/PgnJ | 001697 | BXD87/RwwJ | 007130 |  |  |
| BXD13/TyJ | 000040 | BXD9/TyJ | 000105 |  |  |
| BXD14/TyJ | 000329 | BXD98/RwwJ | 007141 |  |  |
| BXD15/TyJ | 000095 | BXH10/TyJ | 000032 |  |  |
| BXD20/TyJ | 000330 | BXH19/TyJ | 000033 |  |  |
| BXD21/TyJ | 000077 | BXH2/TyJ | 000034 |  |  |
| BXD31/TyJ | 000083 | BXH22/KccJ | 003786 |  |  |
| BXD32/TyJ | 000078 | BXH6/TyJ | 000038 |  |  |
| BXD36/TyJ | 003225 | BXH7/TyJ | 000014 |  |  |
| BXD39/TyJ | 003228 | BXH8/TyJ | 000076 |  |  |
| BXD40/TyJ | 003229 | BXH9/TyJ | 000008 |  |  |
| BXD42/TyJ | 003230 | C3H/HeJ | 000659 |  |  |
| BXD43/RwwJ | 007093 | C57BL/6J | 000664 |  |  |
