# Supplementary material for: A GWAS approach identifies Dapp1 as a determinant of air pollution-induced airway hyperreactivity
Source: PLoS Genet. 2019 Dec 23;15(12):e1008528. doi: 10.1371/journal.pgen.1008528 (PMC6944376; doi:10.1371/journal.pgen.1008528)
Supplement: S2 Table — (DOCX) [file pgen.1008528.s003.docx]

**S2 Table. Association of** **Lead SNP (rs30880385) at GxE Locus on Chromosome 3 with Lung Resistance under Control PBS Conditions.**

|  | **Methacholine Dose (mg.ml^-1^)** | | | | | |
| --- | --- | --- | --- | --- | --- | --- |
|  | **0** | **2.5** | **5** | **10** | **20** | **40** |
| Beta (T allele) | -0.006 | 0.061 | 0.097 | 0.097 | 0.020 | 0.033 |
| *p-value | 8.4x10^-1^ | 1.9x10^-1^ | 6.4x10^-1^ | **2.3x10^-2^** | 6.2x10^-1^ | 3.9x10^-1^ |

Betas are shown for the indicated effect allele as normal inverse transformed values.

*Associations at p-values < 0.05 are shown in bold.
