# Supplementary figures and images for: A GWAS approach identifies Dapp1 as a determinant of air pollution-induced airway hyperreactivity
Source: PLoS Genet. 2019 Dec 23;15(12):e1008528. doi: 10.1371/journal.pgen.1008528 (PMC6944376; doi:10.1371/journal.pgen.1008528)

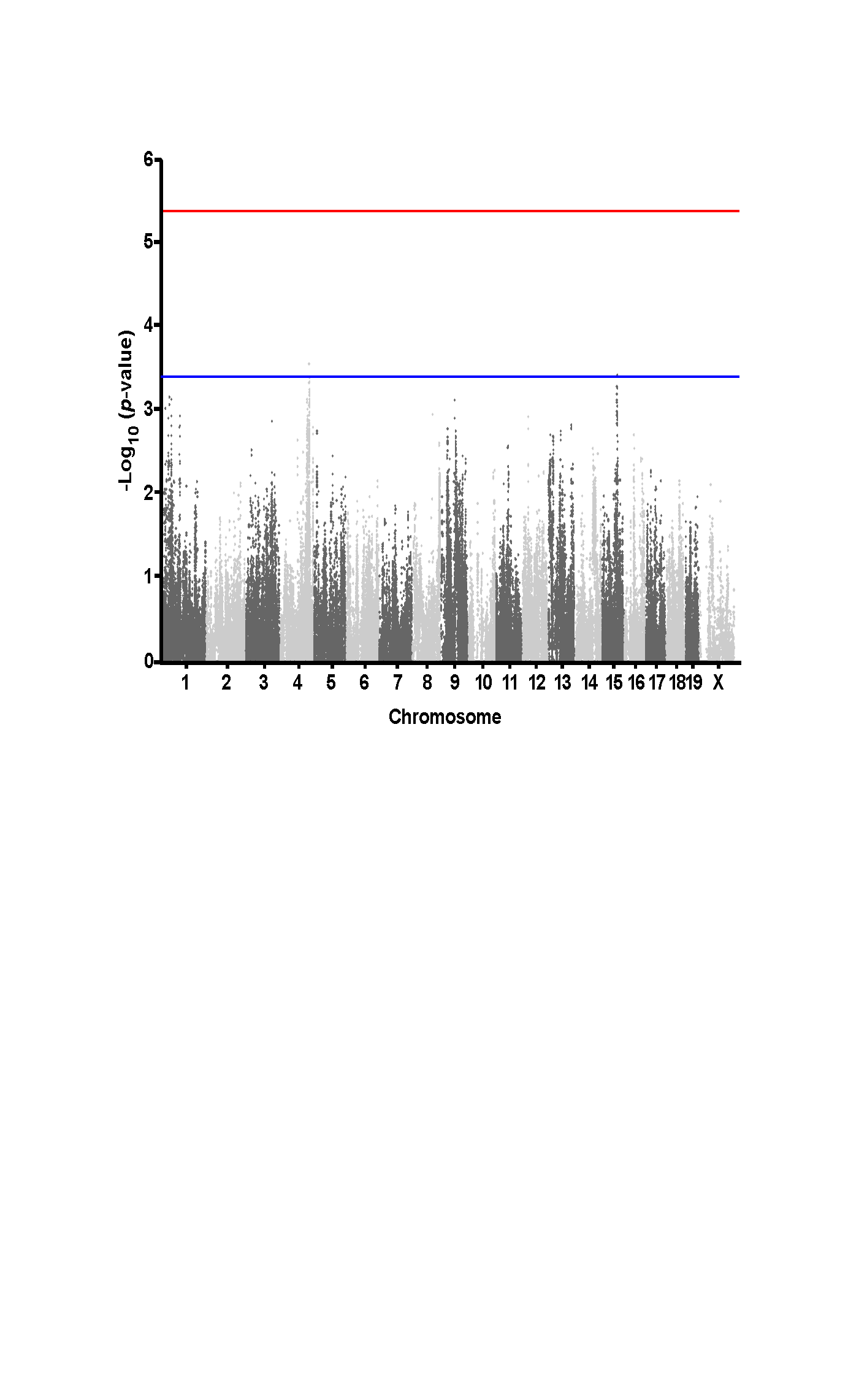

Supplement: S1 Fig — The Manhattan plot shows the results of a GWAS analysis for lung resistance after inhalation exposure to DEP across all methacholine doses, as determined by (AUC) analysis. No locus was genome-wide significant but two loci on chromosomes 4 and 15 exhibited suggestive evidence for association. To be consistent with the phenotype that led to the identification of the chromosome 3 GxE locus, differences (delta, Δ) in AUC values between DEP and PBS (Δ AUCDEP—AUCPBS) were used for this analysis. The GWAS included 203,074 SNPs, whose genomic positions are shown along the x-axis with their corresponding -log10 p-values indicated by the y-axis. The genome-wide thresholds for significant (p = 4.1x10^-6^) and suggestive (p = 4.1x10^-4^) evidence of association are indicated by the horizontal red and blue lines, respectively. (TIFF) [file pgen.1008528.s004.tiff]

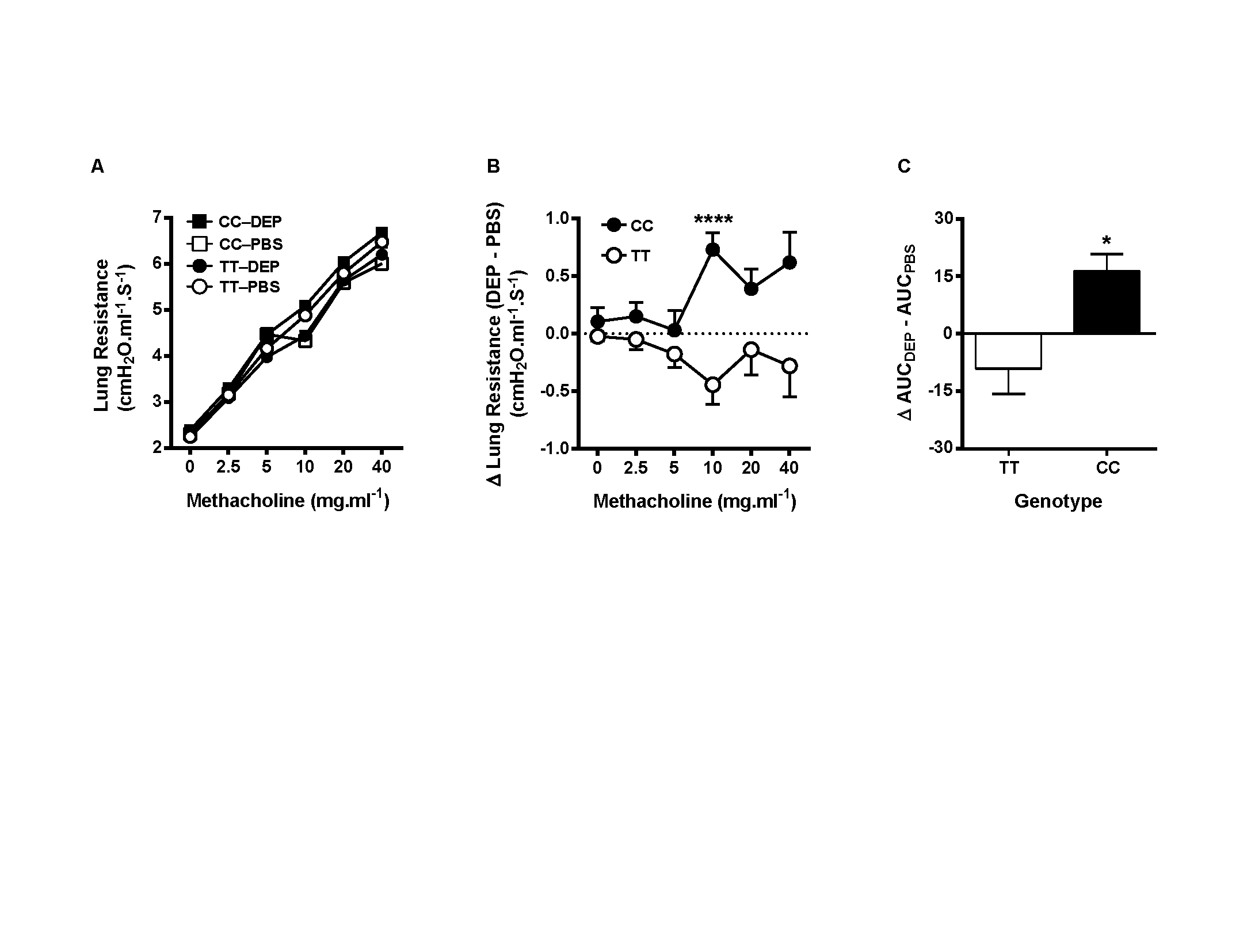

Supplement: S2 Fig — A) Lung resistance is plotted as a function of exposure (PBS or DEP) group and genotypes at rs30880385 across increasing doses of methacholine. B) The difference (delta, Δ) in lung resistance between DEP exposure and PBS control groups is plotted as a function of genotype across increasing doses of methacholine. The Δ value at 10mg/ml methacholine was significantly different (p = 2.5x10^-6^) between strains with CC (n = 25) and TT (n = 72) genotypes at rs30880385 and the basis for identification of the chromosome 3 locus in the GxE GWAS. C) The difference in lung resistance between DEP exposure and PBS control groups across all methacholine doses, as calculated by an area under the curve (Δ AUCDEP—AUCPBS), was also significantly different as a function of genotype. Data are shown as mean ± SE. ****p<0.0001; *p<0.05. (TIFF) [file pgen.1008528.s005.tiff]

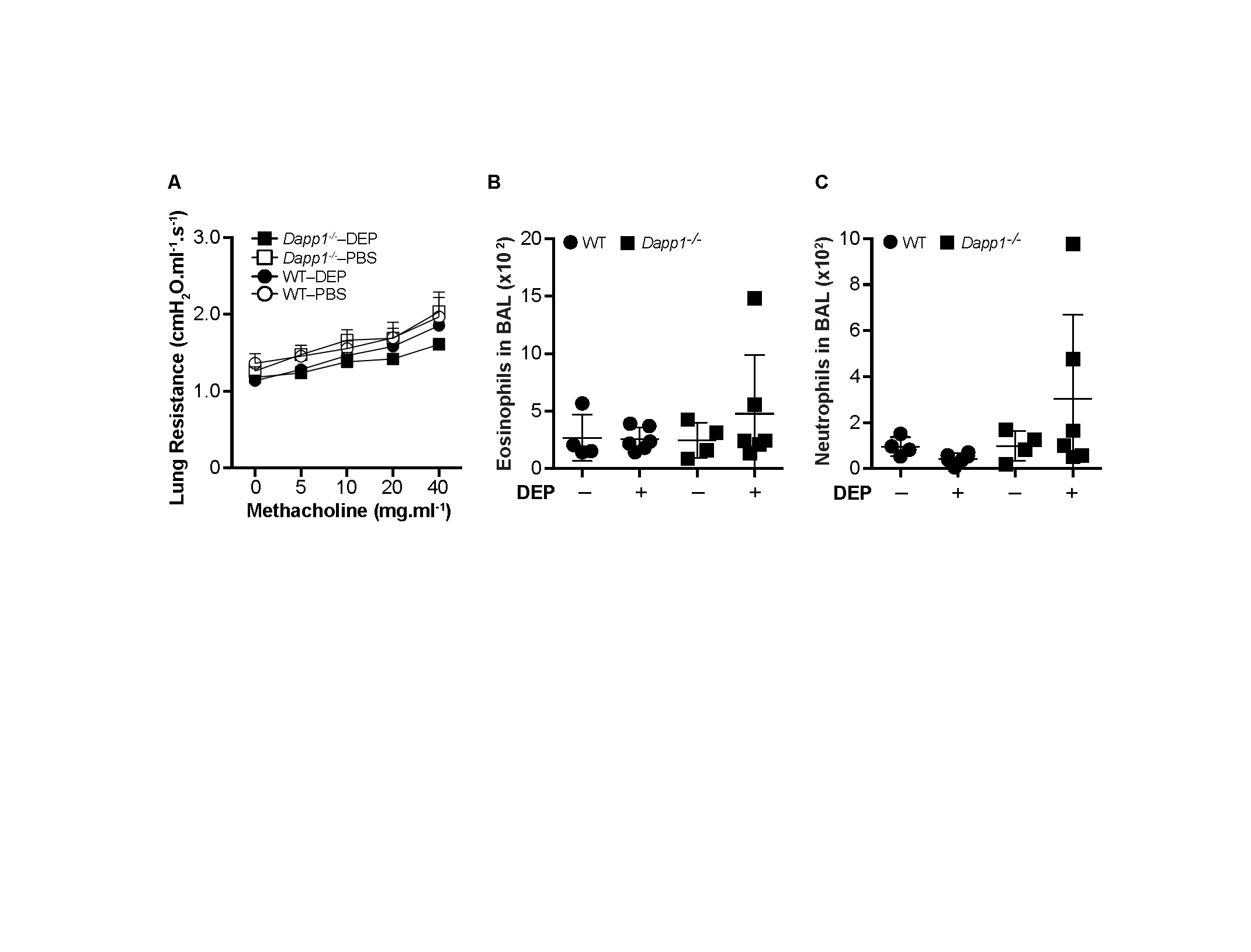

Supplement: S3 Fig — A) Lung resistance after inhalation exposure to DEP is not statistically significantly different between female Dapp1-/- mice compared to control female WT mice but shows a trend for being decreased. There were also no differences in the induction of eosinophilia (B) and neutrophilia (C) in BAL fluid from Dapp1-/- and control WT mice after inhalation exposure to DEP. Mice (n = 4–6 per strain) were first sensitized on day 0 through a 100μl intraperitoneal injection containing 200μg DEP and 25μg HDM + 2.25mg Alum as an adjuvant. On days 7–10, mice were placed in insulated chambers daily for 20mins supplied with ambient air and saturated with aerosolized PBS (as a control) or 200μg DEP, followed measurement of AHR by invasive plethysmography and collection of BAL fluid on day 11. WT control animals were C57BL/6J mice purchased from the Jackson Laboratories. Cell counts in BAL fluid were determined by flow cytometry. Data are shown as mean ± SE. (TIFF) [file pgen.1008528.s006.tiff]
